# Cytokine profile in drug-naïve panic disorder patients

**DOI:** 10.1038/s41398-022-01835-y

**Published:** 2022-02-22

**Authors:** Laiana A. Quagliato, Antonio E. Nardi

**Affiliations:** Laboratory of Panic & Respiration. Institute of Psychiatry, Federal University of Rio de Janeiro. Rua Voluntarios da Patria 190s.722, Rio de Janeiro, 22270-902 Brazil

**Keywords:** Molecular neuroscience, Diagnostic markers

## Abstract

Although accumulating evidence suggests that inflammatory processes play a role in the pathophysiology of mental disorders, few studies have investigated this matter in panic disorder (PD). Furthermore, no studies to date have evaluated cytokine levels in drug-naïve patients with PD. Therefore, little is known about the presence of inflammation at the onset of this disorder. The aim of the present study was to determine the levels of the proinflammatory interleukins IL-1B and IL-2R and the anti-inflammatory cytokine IL-10 in drug-naïve PD patients. Analysis of serum chemokine levels revealed increased proinflammatory activity in the early phase of PD through increased IL-2R and IL-1B levels and a decrease in IL-10 levels in drug-naïve PD patients compared to matched healthy controls. Neurotransmitters and neurocircuits that are targets of inflammatory responses are discussed, followed by an examination of brain–immune interactions as risk factors for PD. This study is the first to identify a proinflammatory cytokine response in drug-naïve PD subjects. These findings indicate that treatments targeting proinflammatory markers may ameliorate anxiety symptoms in PD patients.

## Introduction

Panic disorder (PD) is a chronic mental condition marked by frequent and unexpected panic episodes, as well as persistent concern about panic attacks and their repercussions. PD affects 22.7% of the global population [1]. As a result, PD significantly impacts people’s quality of life and functioning [1]. The neurobiology underlying PD is unknown, and existing pharmaceutical treatments are often ineffective at keeping patients in remission or enhancing their quality of life [2]. A higher frequency of panic attacks has been associated with greater risk of recurrence, severity, and long-term consequences, emphasizing the significance of seeking early treatment [3]. In this context, greater knowledge of the pathophysiology of early-stage PD is crucial for enhancing treatment options and, ultimately, patient outcomes.

The intensity and recurrence of panic attacks have been correlated with changes in immunological markers [4]. Compared with remitted PD, current PD was associated with higher mean levels of inflammatory cytokines, such as IL-6 and IL-1B [4]. Peripherally generated proinflammatory cytokines can pass the blood-brain barrier, and peripheral proinflammatory signals can be actively propagated across the blood-brain barrier through crosstalk between the peripheral and central immune systems [5, 6].

Although inflammatory interleukins have been associated with PD, the pathophysiological mechanisms by which these mediators might contribute to panic symptoms remain unknown. In particular, peripheral cytokines can change amygdala activity and increase anxiety-like behavior [7]. The amygdala, the focal point for fear processing, has been associated with the etiology of PD and panic attacks. Greater inflammation has been linked to greater activation in threat and anxiety-related neurocircuitry, including the dorsal anterior cingulate cortex, insula, and amygdala, based on functional magnetic resonance imaging studies [7–9]

An imbalance between inflammatory and anti-inflammatory cytokines has been reported in several mental health disorders, including PD [4]. However, to date, the cytokine profile of drug-naïve individuals with PD has not been investigated. Thus, the aim of this study was to assess the inflammatory cytokines IL-1B and IL-2R and the anti-inflammatory marker IL-10 in a sample of drug-naïve adults with PD and matched controls.

## Methods

### Study design and participants

This was a cross-sectional study with a matched sample of drug-naïve young adults. For this study, drug-naïve subjects with PD (*n* = 38) were selected. The inclusion criteria for the PD group were as follows: individuals were (1) diagnosed with PD and (2) self-reported no lifetime psychiatric medication use (drug-naïve status) and no current or past psychotherapy. The drug-naïve status was defined as no receipt of psychotropic medications or psychotherapy in a lifetime as demonstrated by universal electronic medical records and confirmed by the patient’s interview. A healthy control group was also recruited (*n* = 38). The inclusion criteria for the healthy control group were no current or previous mental health disorders. Furthermore, potential subjects were excluded for several medical conditions, such as uncontrolled cardiovascular, endocrinological, hematological, hepatic, renal, or neurological disease, autoimmune conditions, chronic infection (i.e., HIV, hepatitis B or C), history of liver abnormalities, or evidence of infection within one month of screening, and their respective treatments, as steroids, antiretroviral therapy, anti-inflammatory, chemotherapy. These conditions and treatments might have confounded study interpretation and were confirmed by medical history (Supplementary Material). Participants were matched by sex, age, and years of education.

### Clinical assessments

PD diagnosis was determined by a structured clinical interview based on the Diagnostic and Statistical Manual of Mental Disorders, Fourth Edition-Text Revision (DSM-IV-TR) administered by a trained psychiatrist or psychologist and independently confirmed by a research psychiatrist. The Hamilton Anxiety Rating Scale (HAM-A) [10], Hamilton Depression Rating Scale (HAM-D) [11], and Panic and Agoraphobia Scale (PAS) [12] were administered to all patients to obtain measures of general psychopathology, and the Clinical Global Impression (CGI) scale [13] was applied to evaluate total symptom severity. All assessments were made by psychiatrists trained in the administration and scoring of the assessments. The height and weight of all participants were recorded for body mass index (BMI) calculation. The procedures were explained, and written informed consent was obtained from participants prior to participation in the study, which was approved by the research ethics committee of the Federal University of Rio de Janeiro. This study was performed in accordance with the ethical standards of the Declaration of Helsinki.

### Interleukins analysis

Blood was obtained in the morning (8 a.m. ± 1 h) in EDTA tubes through a catheter after participants had at least 30 min of rest. Blood was immediately centrifuged (1000 × *g* for 10 min), and serum was removed and stored at −80 °C until the batch assay. Concentrations of the cytokines IL-10 and IL-1B and their soluble receptor, IL-2R, were assessed using the Immulite System (Diagnostic Products Corporation). For further details related to interleukins analysis, please refer to Supplementary Materials.

### Statistical analysis

Statistical analyses were performed using the Statistical Package for the Social Sciences (SPSS) version 26.0. Normality of the distribution of the variables was tested using the Kolmogorov–Smirnov test. The cytokine IL-2R data were normally distributed, but the IL-10 and IL-1B data were not normally distributed. Independent samples t-tests were used for parametric variables, and Mann-Whitney U tests were used for nonparametric variables. Statistical significance was set at *p* < 0.05. Multivariate linear regression models using stepwise methods were performed to evaluate the effect of independent factors on interleukin levels. Multiple linear regression using stepwise methods between the interleukins and several factors related to sociodemographic and physical characteristics (sex, age, educational years, first-degree family member with mental disorder, alcohol consumption, physical activity, BMI, and PAS, HAM-A, HAM-D and CGI scores) as independent variables were performed to assess the relationships of these potentially confounding variables to cytokine levels. Assumptions for linear regression assessed: linearity was checked by a scatter plot, and it depicted no significant outlier. Visual inspection of residual plots did not reveal violations of the normality assumption. Furthermore, multicollinearity was checked, and the Pearson correlation coefficient and variance inflation factor (VIF) found to be < 1. All assumptions were met. The presence of multicollinearity among the independent variables was ruled out by the tolerance level and inflated variance factor (IVF), which was greater than 0.1 and less than 10 for all, respectively. Homoscedasticity was confirmed using a scatter plot of predictors and standardized residuals. The normality of the residuals for each dependent variable was tested using a histogram and Q-Q plot of the standardized residuals. We used the Bonferroni correction to control for multiple comparisons (a total of 16 variables were introduced; thus, the p-value threshold was set at .05/11 = .004).

## Results

The sample was composed of 76 drug-naïve individuals: 38 with PD and 38 healthy controls. The sample was 81.5% female and was successfully matched. There were no significant differences between groups regarding demographic characteristics, as described in Table 1.

**Table 1:** Demographics of study participants.

Demographic (mean and s.d.)	PD (n = 38)	Controls (n = 38)	Difference
Age in years	21.5 (3.0)	19.8 (2.5)	ns
Education in years	10.4 (2.3)	12.6 (1.2)	ns
Sex	32 F: 6 M	30 F: 8 M	ns
BMI	22.92 (2.89)	23.04 (3.22)	ns
PAS [12] mild panic symptoms (<9)- severe symptoms (>28)	26.30 (7.1)		
CGI [13] normal (1)-amongst the most severely ill patients (7).	3.3 (0.69)		
14- item HAM-A [10] mild anxiety (<17)- severe anxiety (>25)	24.25 (13.9)		
17-item HAM-D [11] no depression (<7)-severe depression (≥24)	5.64 (4.07)		
Number of panic attacks in the last month	8.5 (2.5)		
Duration of panic attacks in the last month (range in minutes)	10–60 min		
Severity of panic attacks in the last month (frequency in total cohort)	Mild=31.5% Moderate=42.3% Severe=21% Extremely severe=5.2%		
Age at panic attack onset (year)	20.5 (3.0)		
Duration of untreated panic attacks (months)	12 (2.0)		
Clinical medications in use (frequency in total cohort)	Oral contraceptive pills = 44.7%	Oral contraceptive pills=36.8%	ns

*ns* not significant, *BMI* body mass index, *PAS* Panic and Agoraphobia Scale, *CGI* Clinical Global Impression Scale, *HAM-A* Hamilton Anxiety Rating Scale, *HAM-D* Hamilton Depression Rating Scale.

The multiple regression model did not show any associations between demographic scores and interleukin levels in the PD patients or in the controls. Therefore, interleukin levels were not affected by these potentially confounding variables.

Analysis of serum chemokine levels revealed significantly higher levels of IL-1B (U = 258.5; *p* < 0.001) and IL-2R (t = 5.14; df = 74; *p* < 0.001) in the PD patients than in the healthy controls. Furthermore, IL-10 levels were lower (U = 361; *p* < 0.001) in the PD patients than in the healthy controls (Supplementary Materials).

## Discussion

To our knowledge, this is the first study in which interleukin levels were measured in drug-naïve PD patients and matched controls. Our patient group consisted exclusively of drug-naïve first-episode PD patients who were in the acute phase of their illness. Our results revealed higher levels of the proinflammatory chemokines IL-1B and IL-2R and lower levels of the anti-inflammatory cytokine IL-10 in drug-naïve PD patients than in healthy control subjects.

Our findings suggested increased proinflammatory activity in the early phase of PD through increased IL-2R and IL-1B activity and a decrease in IL-10 levels. The differences between proinflammatory interleukins and anti-inflammatory interleukins might be related to microglia [14]. Microglia, the brain’s endogenous immune cells, can switch from a surveillance mode to various levels of activation or reactivity in response to environmental stimuli (e.g., stranger and danger signals) [15]. M1, the classic activated state of microglia, releases proinflammatory mediators such IL- 6, IL-8, IL-1, IL-2R, and reactive oxygen species (ROS) and has been implicated in neurotoxicity [16]. The anti-inflammatory features of the M2 phenotype, on the other hand, stimulate tissue remodeling and repair by releasing high quantities of IL-4 and IL-10 [17]. Evidence points toward chronic activation of microglia in several mental health disorders, such as schizophrenia, bipolar disorder, depression, and autism [18, 19]. Chronic activation of microglia leads to increased production of cytokines and an imbalance between the pro‐inflammatory and anti‐inflammatory states [20]. Considering our findings, it is reasonable to hypothesize that in PD patients, microglia might be chronically activated and producing inflammatory interleukins.

**Fig. 1:**
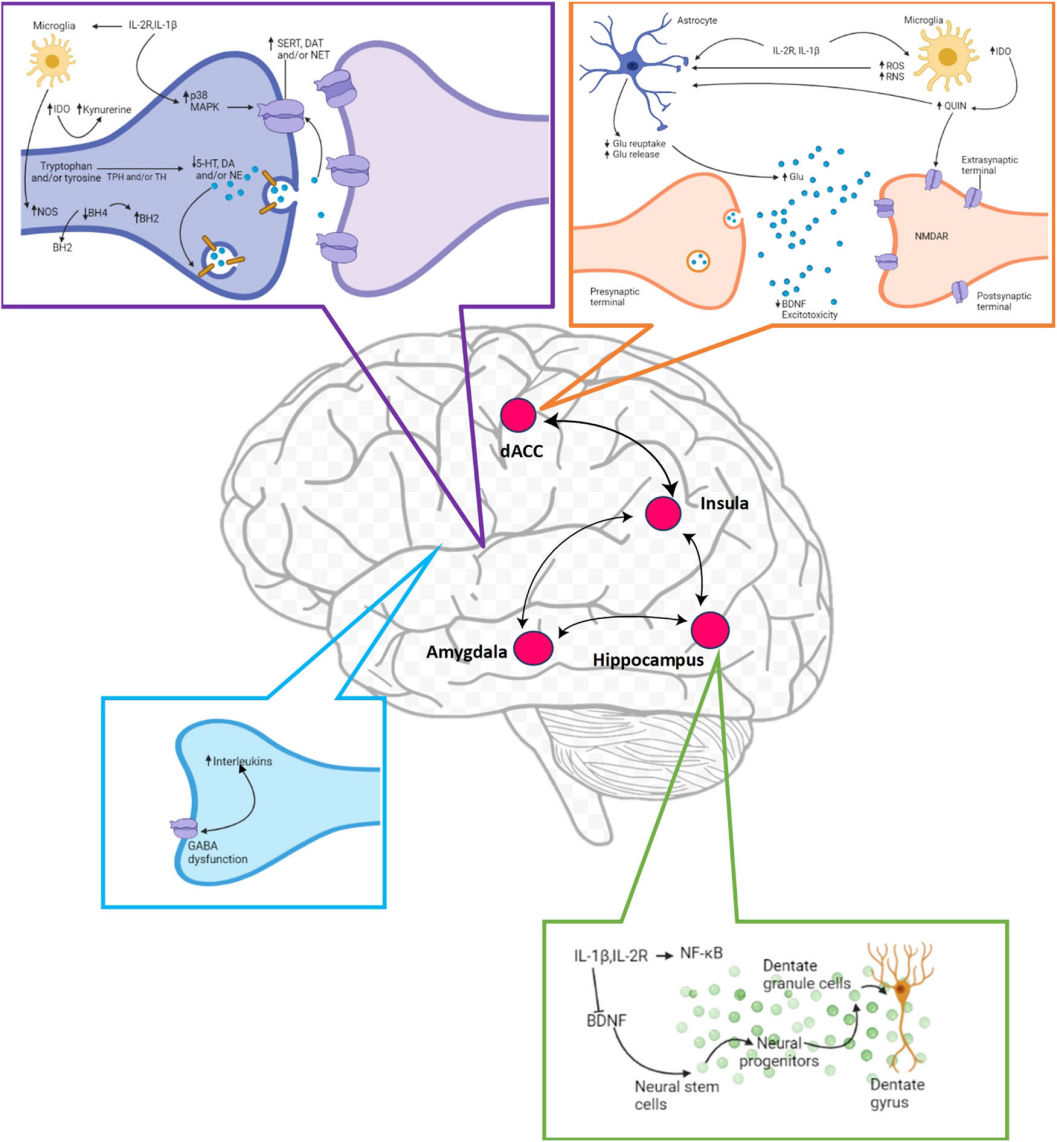
Inflammatory responses in the brain can affect molecular pathways influencing neurotransmitter systems that can ultimately affect neurocircuits relevant to PD. Proinflammatory cytokines such as interleukin 1B (IL-1B) and IL-2R can decrease the availability of monoamines such as serotonin (5-HT), dopamine (DA), and noradrenaline (NE) by enhancing the expression and function of presynaptic reuptake pumps for 5-HT, DA, and NE by activating mitogen-activated protein kinase (MAPK) pathways and by reducing enzymatic cofactors such as tetrahydrobiopterin (BH4). This cofactor is sensitive to cytokine-induced oxidative stress and is involved in the production of nitric oxide (NO) through NO synthase (NOS). The inflammatory interleukins IL-1B and IL-2R can also reduce relevant monoamine precursors by activating the enzyme indoleamine 2,3-dioxygenase (IDO), which converts tryptophan, the primary precursor for serotonin, into kynurenine. Activated microglia can convert kynurenine to quinolinic acid (QUIN), which binds to the glutamate (Glu) N-methyl-d aspartate receptor (NMDAR). This, the combination of cytokine-induced reductions in astrocytic Glu reuptake and stimulation of astrocyte Glu release can lead to excessive Glu, an excitatory amino acid neurotransmitter. Excessive Glu, especially when binding to extrasynaptic NMDARs, can then lead to reduced brain-derived neurotrophic factor (BDNF) levels and excitotoxicity. Inflammation-related effects on BDNF can also affect neurogenesis and long-term potentiation. Furthermore, GABA dysfunction might not properly inhibit inflammatory responses. Cytokine effects on neurotransmitter systems can activate circuits, including the amygdala, hippocampus, dorsal anterior cingulate cortex, and insula, that regulate anxiety, arousal, alarm, and fear. BH2 dihydrobiopterin, DAT dopamine transporter, NET noradrenaline transporter, NF-κB nuclear factor-κB, SERT serotonin transporter, TH tyrosine hydroxylase, TPH tryptophan hydroxylase, ROS reactive oxygen species, RNS reactive nitrogen species, IDO indoleamine 2,3-dioxygenase, Quin quinolinic acid, Glu glutamate, dACC dorsal anterior cingulate cortex.

Given the pivotal role of neurotransmission in anxiety disorders, it is important to understand how inflammation and inflammatory cytokines affect the monoamines serotonin, noradrenaline, and dopamine, the excitatory amino acid glutamate, and the inhibitory neurotransmitter gamma-aminobutyric acid (GABA). There are several mechanisms (Fig. 1) through which inflammatory cytokines can cause reduced synaptic availability of monoamines, which is believed to be a key factor in the pathophysiology of PD [21]. In animal models, for example, IL-1B induction of p38 mitogen-activated protein kinase (MAPK) increased the expression and function of serotonin reuptake pumps, resulting in decreased synaptic availability of serotonin and anxiety-like behavior [22]. Inflammatory cytokines, by generating reactive oxygen and nitrogen species, have also been reported to reduce the availability of tetrahydrobiopterin (BH4), a critical enzyme cofactor in the production of all monoamines that is especially sensitive to oxidative stress [23]. Indeed, in patients treated with the inflammatory cytokine IFN-alpha, BH4 levels in the cerebrospinal fluid were found to be negatively correlated with inflammatory interleukin levels in the cerebrospinal fluid [24, 25].

Inflammatory interleukins have also been associated with alterations in tryptophan metabolism, the primary amino acid precursor of serotonin. The indoleamine-2,3-dioxygenase (IDO) enzyme, which catalyzes the rate-limiting step in the synthesis of kynurenine from tryptophan, can be induced by proinflammatory cytokines (Capuron et al., 2011). In central and peripheral immune-competent cell types, inflammatory cytokines, such as IL-1B and IL-2R, have been shown to increase IDO expression [6]. Tryptophan can be degraded into kynurenine by activation of these cell types, which can lead to PD symptoms by reducing the availability of the necessary precursor for serotonin synthesis, causing disruption of serotonergic neurotransmission [26]. Furthermore, activated microglia in the brain can convert kynurenine to the neurotoxic metabolite quinolinic acid [26]. Quinolinic acid stimulates glutamate release, blocks glutamate absorption by astrocytes, and directly activates glutamate receptors (N-methyl-d-aspartate (NMDA) receptors) [27]. The direct effects of proinflammatory cytokines on glutamate metabolism, which include decreasing the expression of astrocyte glutamate reuptake pumps and stimulating astrocytic glutamate release, converge with the effects of quinolinic acid on glutamate metabolism, ultimately contributing to excessive glutamate both within and outside the synapse [27]. Increased excitotoxicity and decreased synthesis of brain-derived neurotrophic factor (BDNF) result from glutamate binding to extrasynaptic NMDA receptors [28]. BDNF promotes neurogenesis, an important prerequisite for an antidepressant response [29]. However, in stress-induced animal models of anxiety, this molecule has been shown to be reduced by IL-1B and IL-2R and their downstream signaling pathways, including NF-κB [30].

An increase in inflammatory cytokine levels could also be linked to PD through GABA deficit dysfunction [4]. GABAA receptors inhibit inflammatory cytokine expression and play an important anti-inflammatory role [31]. Evidence has shown that this receptor is dysfunctional in PD patients, which could contribute to PD pathophysiology [32]. Endogenous GABA tonically inhibits inflammatory interleukins, such as IL-6 and IL-1B, through GABA receptors [31]. Therefore, dysfunction of the GABAergic system has the great potential to not properly inhibit inflammatory responses, contributing to PD symptoms.

Our results are in accord with previous studies that suggested that changes in inflammatory mediators may be associated with the pathophysiology of PD [4]. However, several questions remain to be answered. The physiological role of chemokines in the central nervous system (CNS), as well as the specific mechanisms of regulation and balance of different inflammatory mediators in health and disease, are currently being investigated [33]. Therefore, much of the role of interleukins in the CNS is currently unknown. This study has strengths and limitations that should be considered when interpreting the results. The diagnostic interviews of both patients and controls were performed using the same protocol. In addition, blood samples were obtained between 8 and 9 a.m., minimizing circadian differences in cytokine levels potentially related to cortisol release. On the other hand, limitations inherent to cross-sectional studies (and thus also present here) preclude conclusions about causality.

In summary, our study is significant in that it is the first to provide evidence for an increase in the inflammatory mediators IL-1B and IL-2R and a decrease in anti-inflammatory molecules, such as IL-10, in nonmedicated PD patients. If the current findings are confirmed in future studies, it could pave the way for a new era in PD treatment options and neuroscience research in general. Such complexities should be taken into account when developing drugs that act on chemokines and could be used to treat PD.

## Supplementary information

Supplementary material
