# Supplementary material for: Cytokine profile in drug-naïve panic disorder patients
Source: Transl Psychiatry. 2022 Feb 22;12:75. doi: 10.1038/s41398-022-01835-y (PMC8863842; doi:10.1038/s41398-022-01835-y)
Supplement: Supplementary file 1 — Supplementary material [file 41398_2022_1835_MOESM1_ESM.doc]

**Supplementary Methods**

**Participants**

An a priori power analysis using previous cytokine data confirmed that a minimum of 36 participants per group would give the current study 80% power to detect a significant difference between groups if one existed^1^ . Subjects were excluded for a number of medical conditions that might confound study interpretation as confirmed by medical history. Individuals were excluded for uncontrolled cardiovascular, endocrinological, hematological, hepatic, renal, or neurological disease, autoimmune conditions, chronic infection (i.e., HIV, hepatitis B or C), history of liver abnormalities, or evidence of infection within one month of screening. Participants were evaluated using SCID-IV^2^ and excluded for a history of schizophrenia; active psychotic or depressive symptoms of any type; substance abuse and/or dependence within the past 6 months; an active eating disorder or obsessive compulsive disorder; and/or a score of less than 28 on the Mini-Mental State Examination^3^. Blood was collected at the Institute of Psychiatry at one screening visit for an analysis of interleukin levels.

**Laboratory assays: measurement cytokine levels**

Concentrations of cytokines and their soluble receptors were assessed using Immulite System (Diagnostic Products Corporation)^4^. This technology is based on a solid phase two-site chemiluminescent enzyme immunometric assay. The solid phase, a polystyrene bead, is coated with either a monoclonal specific antibody or an anti-ligand. Patient serum and alkaline phosphatase-conjugated monoclonal antibody or, depending on the technique, a ligand-labeled antibody is incubated for 30 to 60 min at 37 °C. Unbound conjugate is then removed by a centrifugal wash (x3), after which a chemiluminescent substrate (a phosphate ester of adamantyl dioxetane) is added, and the test unit is incubated for a further 10 minutes. The chemiluminescent substrate undergoes hydrolysis in the presence of alkaline phosphatase to yield an unstable intermediate with an emission of light. The bound complex, and thus also the photon output, as measured by the luminometer, is proportional to the concentration of cytokine in the sample. For each cytokine calibration, a master curve is constructed by the manufacturer using a material calibrated against the National Institute for Biological Standards and Control standards ^3^ . The analytical sensitivities of the IL-1B, IL-2R, and IL-10 assays were 1.5 pg/ml, 10 U/ml, and 1.0 g/ml, respectively, and the calibration range was up to 1000 ng/L for the three assays. Immulite cytokine controls were included in each analytical run. The intra-assay and interassay precision performances of the assays were determined on 10 replicates in a single run and in 20 different runs, respectively, and yielded intra- and interassay coefficients of variation for each cytokine of 5.8% and 1.1–6.9% for IL-10, 9% and 4–8.6% for IL-1B, and 6.5% and 2.3–7.9% for IL-2R, respectively.

**Supplementary Results**

| **Table 1. Serum cytokine levels in the study sample. Values are mean rank or mean with the standard deviation in parentheses.** | | | | | |
| --- | --- | --- | --- | --- | --- |
| **Interleukins** | **Patients** | **Controls** | **Statistic** | **p- value** | **Effect size** |
| IL-2R | 54.44 (13.62) | 35.84 (17.62) | t= 5.14; df =74 | <0.001 | 1.18 |
| IL-10 | 29 | 48 | U = 361 | <0.001 | 0.19 |
| IL-1B | 50.7 | 26.3 | U= 258.5 | <0.001 | 0.31 |

REFERENCES

1. Petrikis, P., Voulgari, P. V., Tzallas, A.T., Archimandriti, D.T., Skapinakis, P., Mavreas, V., 2015. Cytokine profile in drug-naïve, first episode patients with psychosis. J. Psychosom. Res. 79, 324–327.
2. First, M. B., Spitzer, R. L., Gibbon, M., & Williams, J. B. W., 1996. Structured clinical interview for DSM-IV axis I disorders, clinician version (SCID-CV). Washington, DC: American Psychiatric Press.
3. Folstein, M.F., Robins, L.N., Helzer, J.E., 1983. The Mini-Mental State Examination. Arch Gen Psychiatry 40(7), 812.
4. Berthier, F., Lambert, C., Genin, C., Bienvenu, J., 1999. Evaluation of an automated Immunoassay method for cytokine measurement using the Immulite Immunoassay system. Clinical chemistry and laboratory medicine 37(5), 593-599.
